# Urban park qualities driving visitors mental well-being and wildlife conservation in a Neotropical megacity

**DOI:** 10.1038/s41598-024-55357-2

**Published:** 2024-02-28

**Authors:** Jéssica Francine Felappi, Jan Henning Sommer, Timo Falkenberg, Wiltrud Terlau, Theo Kötter

**Affiliations:** 1 Center for Development Research, University of Bonn, Genscherallee 3, 53113 Bonn, Germany; 2 GeoHealth Centre, Institute for Hygiene and Public Health, University of Bonn, Venusberg Campus 1, 53105 Bonn, Germany; 3 International Centre for Sustainable Development, Bonn‐Rhein‐Sieg University of Applied Sciences, Grantham-Allee 20, 53757 Sankt Augustin, Germany; 4 Urban Planning and Land Management Group, Institute of Geodesy and Geo-Information, University of Bonn, Nussallee 1, 53115 Bonn, Germany

**Keywords:** Biodiversity, Ecosystem services, Urban ecology, Public health

## Abstract

Green infrastructure has been widely recognized for the benefits to human health and biodiversity conservation. However, knowledge of the qualities and requirements of such spaces and structures for the effective delivery of the range of ecosystem services expected is still limited, as well as the identification of trade-offs between services. In this study, we apply the One Health approach in the context of green spaces to investigate how urban park characteristics affect human mental health and wildlife support outcomes and identify synergies and trade-offs between these dimensions. Here we show that perceived restorativeness of park users varies significantly across sites and is mainly affected by safety and naturalness perceptions. In turn, these perceptions are driven by objective indicators of quality, such as maintenance of facilities and vegetation structure, and subjective estimations of biodiversity levels. The presence of water bodies benefited both mental health and wildlife. However, high tree canopy coverage provided greater restoration potential whereas a certain level of habitat heterogeneity was important to support a wider range of bird species requirements. To reconcile human and wildlife needs in green spaces, cities should strategically implement a heterogeneous green infrastructure network that considers trade-offs and maximizes synergies between these dimensions.

## Introduction

Mental health has become a growing concern worldwide after the onset of the COVID-19 pandemic. Initial estimates show up to 28% increase in cases of mental disorders^1^. Globally, in comparison to their rural counterparts, urban dwellers show higher rates of anxiety, mood, and psychotic disorders, a phenomenon called urban psychological penalty^2^, which is partially associated with characteristics of the physical environment^3,4^. Nature has increasingly been acknowledged for its beneficial effect on human health and well-being and, more recently, “green prescription” has emerged as a nature-based health intervention typically designed to tackle non-communicable diseases and mental health issues through the exposure of patients to natural environments^5^. Even in anthropic environments, studies have demonstrated that urban nature (i.e. urban green infrastructure) affects mental health and well-being in terms of stress reduction^6^, mental restoration^7^, life satisfaction^8^, lower depression risk^9^ and anxiety prevalence^10^. However, evidence on the impact of urban green infrastructure quality, in terms of green space characteristics, in the provision of mental health benefits is still limited and dominated by findings from Europe, North America, and Asian countries^11–13^.

Urban green infrastructure can also play a relevant role in biodiversity conservation when sustaining significant plant and animal species and functioning as stepping stones and corridors for wildlife^14^. Support to urban wildlife, defined as non-domestic animals located in human-dominated and non-agricultural areas^15^ is affected by green space quality as aspects such as area, habitat diversity, and tree species richness have already been reported as predictors of animal diversity^16,17^. The knowledge of how animals respond to different characteristics of urban green infrastructure is essential to guide minimal requirements for green space design and management, avoiding the collection of “green deserts” dominated by mowed lawn areas that do not promote wildlife conservation^18^.

Beyond the multifold benefits of green spaces to humans and biodiversity, the effect of biodiversity levels on human health and well-being outcomes has also been explored. Although studies have found mixed results, there is some initial evidence of a positive relationship^19–21^. Nonetheless requirements for human use and wildlife support may differ and a green space might not fulfill the demands for both dimensions at the same time. With the constant pressure on remaining green areas due to the increasing urban population and associated household and infrastructure developments, there is a need for multifunctional green spaces that maximize synergies and manage trade-offs between people and nature. For such, interdisciplinary studies on the role of green space quality on outcomes for both dimensions are necessary.

In this study, we examine the indirect effect of urban park qualities on the perceived restorativeness of users, as well as their direct effect on wildlife support metrics. We hypothesize that among synergies, some park characteristics, especially vegetation-related, may show opposite effects on humans and animals due to, for instance, local safety issues.

## Theoretical framework

The relationships explored here are based on the One Health framework for urban green spaces^12^, which aims at understanding the interlinkages between the green space quality (environmental health), users’ mental health and well-being (human health), and support to urban wildlife (animal health). The focus is to investigate whether green spaces’ characteristics affect outcomes for each dimension and, especially, to identify potential synergistic effects to be maximized and trade-offs to be managed in order to inform the design and management of multifunctional green spaces.

**Figure 1:**
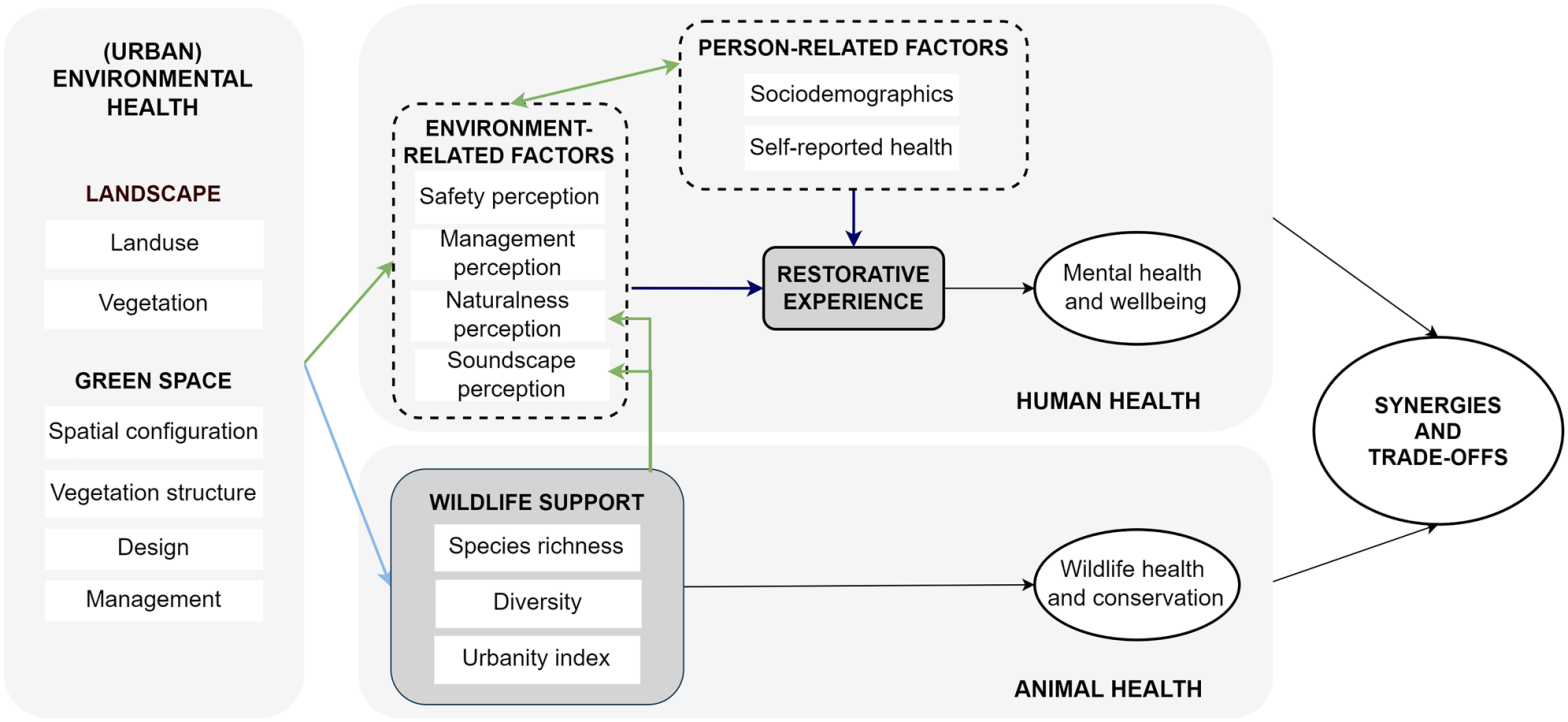
Theoretical framework depicting the relationships investigated in this study. Modified from Felappi et al.^12^. Colors refer to the three steps of the analytical strategy: dark blue—first, green—second, and light blue—third step. A complete description of factors and interlinkages can be found in the original paper.

For this study, we selected urban parks as the green space type as they are freely accessible to the general population. From the multiple pathways linking green spaces to human mental health and well-being^22,23^ we focus on the restorative experience. According to the Attention Restoration Theory, it is the capacity to recover the directed attention that may become depleted in daily life and may lead to impaired performance and stress response^24^. Perceived restorativeness is a known mediator in the relationship between settings experience and well-being outcomes^19^, and it is usually associated with natural environments^25^. Characteristics of the setting may indirectly affect its restorative potential through users’ perceptions of how natural the place looks like (naturalness), the pleasantness of sounds they can hear (soundscape), the maintenance of facilities and vegetation (management), and how safe they feel during the visit (safety)^12^ (Fig. 1). Similarly, several environmental characteristics are known to potentially affect the capacity of green spaces to support urban wildlife populations (wildlife support)^26^. Support to wildlife populations can be assessed through metrics such as diversity estimates^12^.

We investigated these relationships in a case study conducted in a megacity of the Global South, São Paulo, Brazil. First, we explore how people’s perceptions of the setting (i.e., environment-related factors) contribute to their restorative experience and how they are associated with objective indicators of the setting’s quality and wildlife support (Fig. 1). Users’ personal characteristics (i.e., person-related factors) are also considered to have an impact on the individual restorative experience. Second, we analyze how the same objective indicators of the setting’s quality affect wildlife support metrics. Finally, we identify and discuss potential synergies and trade-offs comparing the results for each dimension.

## Results

A total of 994 questionnaires were collected in 20 sites. The sex proportion in our sample was different than the general population of São Paulo (52.6% female^27^), X^2^ (1, N = 994) = 6.73, p ≤ 0.01), with females less represented than males (Table 1). The higher proportion of respondents belonged to the range between 25 and 34 years old and reported having household income lower than two minimum wages.

**Table 1:** Sociodemographic aspects of the sample. MW refers to minimum wage, which was equivalent to 998,00 Brazilian reais in 2019.

	n	%
Sex		
Female	486	48.9
Male	508	51.1
Age		
18–24	168	16.9
25–34	266	26.8
35–44	213	21.4
45–54	156	15.7
55–64	122	12.3
65+	69	6.9
Family monthly income		
< 2 MW	353	35.5
2–4 MW	285	28.7
4–10 MW	217	21.8
10–20 MW	66	6.6
> 20 MW	22	2.2
Did not answer	51	5.1

**Figure 2:**
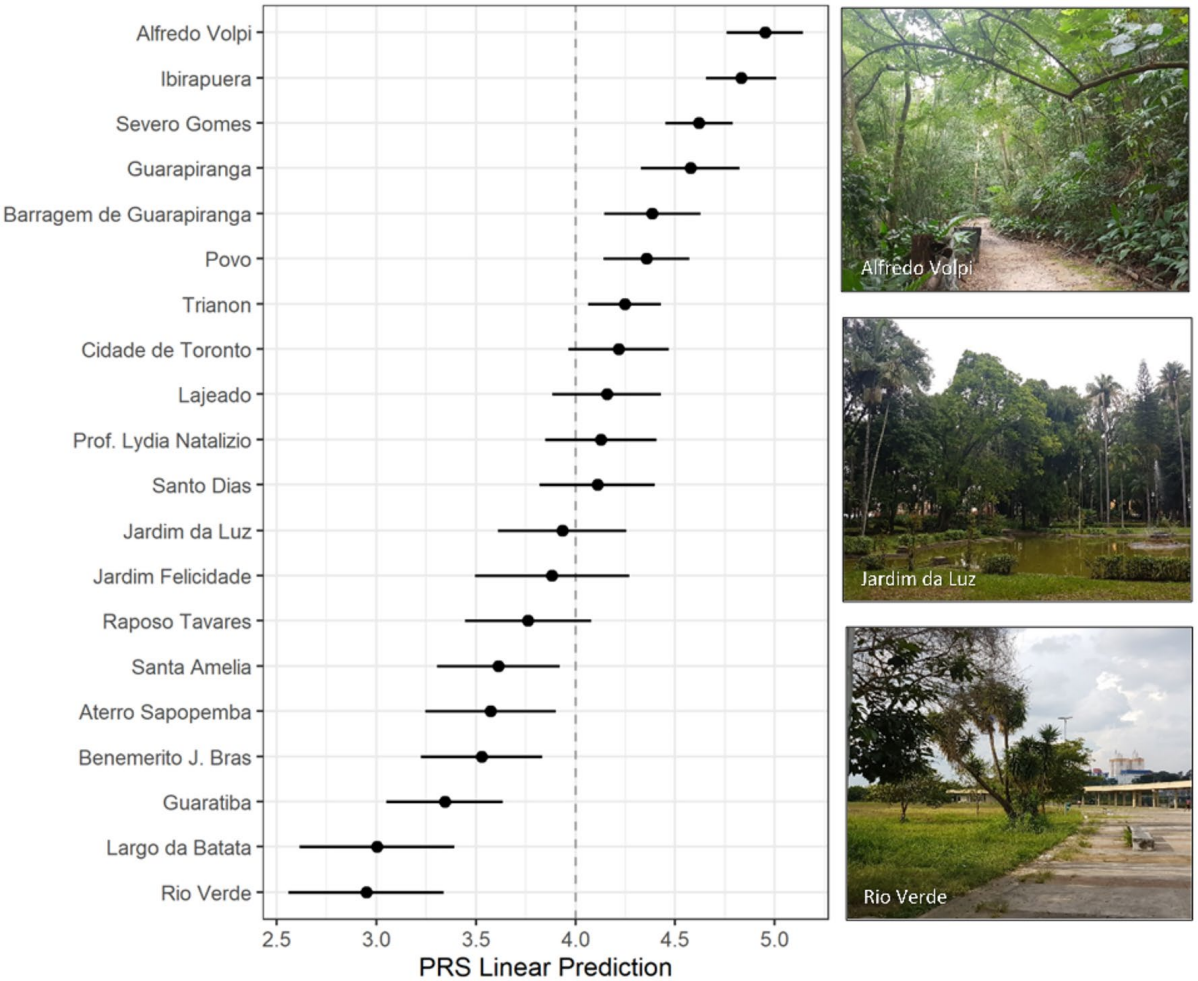
Adjusted predictions of perceived restorativeness scores (PRS) for each park and 95% confidence intervals. The dotted line represents the mean perceived restorativeness score of the total sample. The pictures provide an idea of the different parks’ configuration and represent Alfredo Volpi (upper right), Jardim da Luz (middle), and Rio Verde (bottom).

The Perceived Restorativeness Scale had a mean score of 4.02 (SD = 1.13) and was sensitive to park conditions, showing statistically significant differences in PRS score among sites (Fig. 2). The predicted PRS score values of Rio Verde park and Largo da Batata square (the negative control site) are lower than all others (p < 0.04) except Guaratiba park (p = 0.112 and p = 0.169, respectively). Alfredo Volpi park shows the highest value in comparison to all parks (p < 0.02), not differing (p = 0.360) only in relation to Ibirapuera park (the positive control site).

Although the overall means of perceived naturalness, soundscape, and management scores were similar (3.09, SD = 1.34; 3.31, SD = 1.37; 3.31, SD = 1.66, respectively), they significantly varied among sites too (Supplementary Figs. 1–3). Alfredo Volpi park achieved higher mean perception scores in soundscape and naturalness dimensions (Supplementary Figs. 1 and 3), while Povo park had the highest mean score in management perception (Supplementary Fig. 2). On the other hand, Largo da Batata square had the lowest mean score in soundscape and naturalness perceptions, and Rio Verde park scored lowest in management perception. Overall mean safety perception was 3.48 (SD = 1.8), with the lowest value found in Rio Verde and the highest value in Alfredo Volpi.

## Personal factors and perceptions affecting restorativeness

The first structural equation model (full model) showed moderate fit indices (model 1, Supplementary Table 1) and was followed by optimized models excluding the control variable age (model 2), which had an irrelevant effect on perceived restorativeness, and the exclusion of the additional non-significant pathways of health and stress perceptions (model 3, Supplementary Table 1).

**Figure 3:**
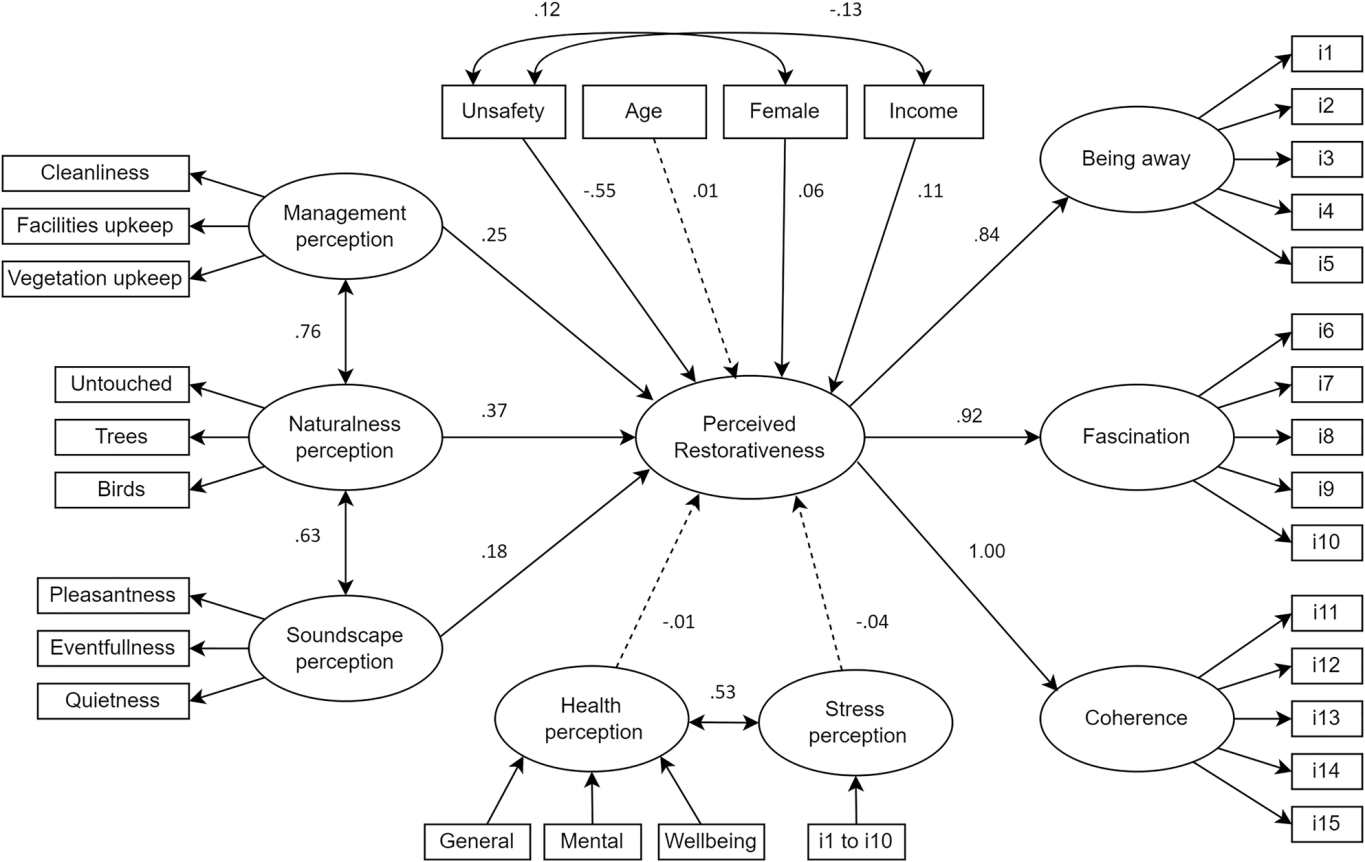
Path diagram of the perceptions and control variables affecting perceived restorativeness (full model, model 1 in Supplementary Table 1) with standardized coefficients. Dotted lines depicture pathways that were statistically non-significant (p > 0.05). Regression estimates within the measurement models were omitted in this picture for simplification.

Safety perception was the variable with the largest standardized regression weight in the model, meaning that feeling unsafe in the setting had the strongest effect on perceived restorativeness, affecting it negatively (Fig. 3, Supplementary Table 2). Feeling unsafe was positively correlated with being female and lower household income (less than 4 minimum wages). Higher household income and being female affected positively the perceived restorativeness, however, with weaker effect sizes than setting perceptions variables.

Respondents’ evaluation of the park as close to original nature and biodiverse (naturalness perception) was the most relevant setting perception, followed by management perception, and soundscape perception (Fig. 3, Supplementary Table 2). Naturalness perception was also correlated with management and soundscape perceptions. Self-reported health condition, stress level, and age were not relevant in the model due to small effect sizes and non-significant p-values.

## Park qualities effect on setting perceptions

As a preliminary analysis, we explored whether people correctly perceived the actual number of bird and tree species. The correlation between the actual number of bird and tree species and respondents’ estimation was low (r = 0.214, p = 0.000; r = 0.40, p = 0.000, respectively). Taking birds species richness estimation as an example, when respondents (N = 888) were asked about the range of bird species present in the park, 85.7% (N = 761) perceived fewer species, and only 8.1% (N = 72) perceived the correct range of species. More than half of respondents (55.5%, N = 493) estimated the presence of up to 15 species and 77.5% of up to 30 species, whereas 15 study sites reportedly have bird richness higher than 30 species with reported numbers for all study sites between 17 and 223 species per park. These results made us further investigate whether the respondents’ estimation of bird and tree richness instead of the actual number of species would produce different outcomes in the setting perceptions models. For that, we compared and reported both models below.

**Figure 4:**
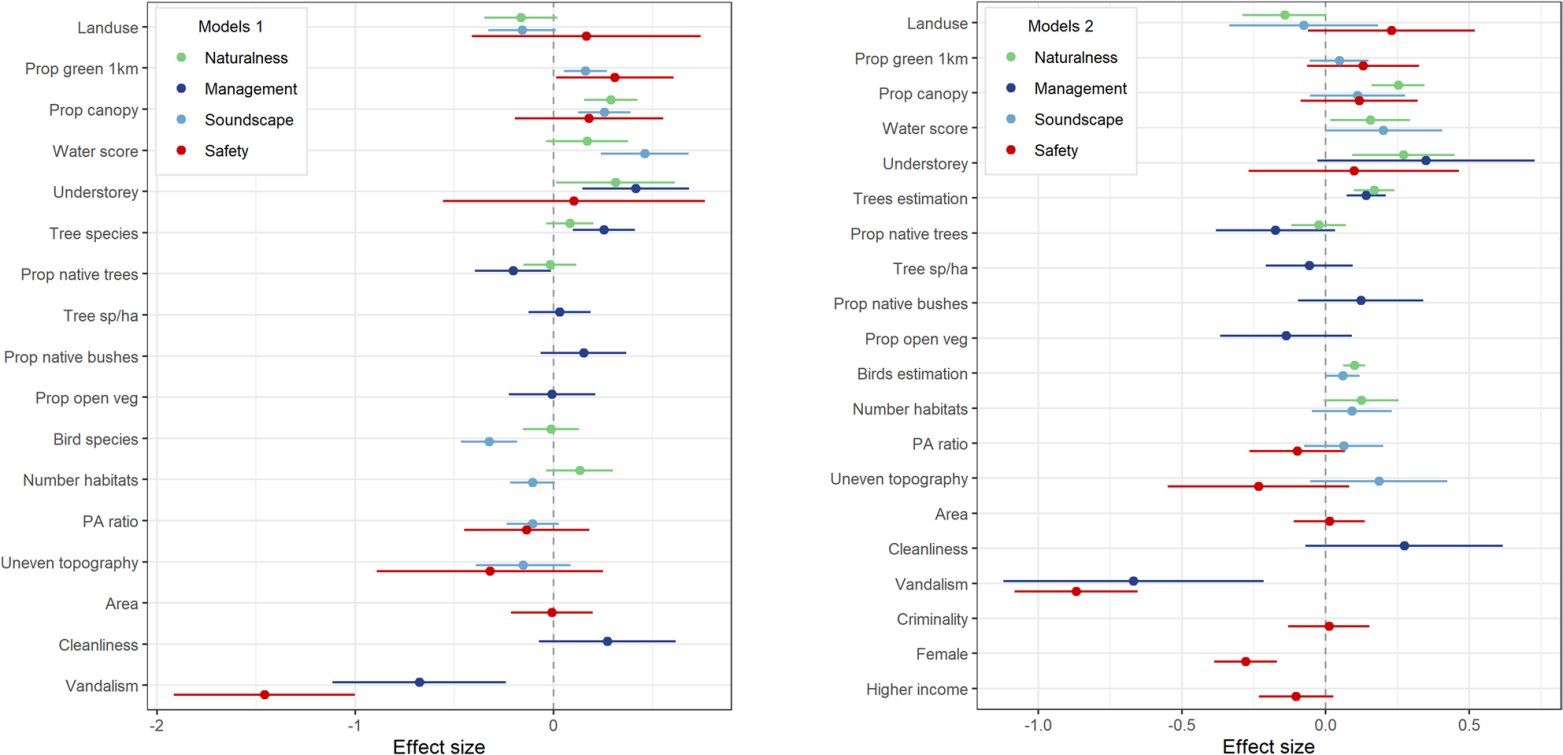
Standardized coefficients and 95% confidence interval of each variable included in the final models of setting perceptions (naturalness, management, and soundscape) and safety perception. The plots represent the models with objectives variables only (models 1, left side), and the models replacing biodiversity perception in the setting perceptions and including control variables in safety perception (models 2, right side). Variables with confidence intervals (bars) that do not cross 0 are statistically significant. See Table 2 for definition of variables.

In the naturalness perception model with objective predictors, only the proportion of tree canopy (p = 0.000) and the presence of understorey vegetation (p = 0.040) were significant predictors, showing positive and similar effect sizes (Fig. 4). The concurrent model (model 2, Fig. 4), which replaced the actual number of bird and tree species with the estimated mean number by the respondent, yielded a lower AIC value (2567.097 versus 2592.133). In this improved model, the proportion of canopy (p = 0.000) and understorey presence (p = 0.003) remained as significant factors with higher effect sizes on naturalness perception however, birds (p = 0.000) and trees (p = 0.000) species estimations, as well as water score (p = 0.028), were also statistically significant predictors but with lower effect sizes.

Based on the best model (model 2), we further explored the effect of different levels of canopy coverage on naturalness perception. Keeping all covariates constant, our model indicates that the mean predicted value of naturalness starts to increase further than the overall mean (3.09) from 60% of canopy coverage (3.19, 95% CI 3.10–3.27), achieving the higher predicted mean at near 100% coverage (3.53, CI 3.39–3.67). In terms of water elements, sites without or with non-accessible water bodies show a predicted mean value lower than the naturalness perception overall mean (3.05, CI 2.84–3.25), whereas the presence of accessible artificial water bodies (3.27, CI 3.18–3.35) and, especially, access to natural water bodies (3.49, CI 3.26–3.71) increased the predicted values.

Respondents’ management perception was negatively affected by the presence of signs of vandalism (p = 0.002), followed by positive associations with the presence of understorey vegetation (p = 0.003) and tree species richness (p = 0.001) (model 1, Fig. 4). The proportion of native trees showed a lower and negative effect size (p = 0.037). In the alternative model (model 2), only vandalism (p = 0.004) and the estimation of tree richness (p = 0.000) were significant predictors, the first with the strongest effect size. The second model yielded a lower AIC value (3151.417) than model 1 (3178.294).

The objective model for soundscape perception resulted in four significant predictors. Water score (p = 0.000) and bird species number (p = 0.000) had the strongest effect on the perception of soundscape however, the latter showed a negative relationship. The proportion of canopy (p = 0.000) and proportion of green within 1 km radius (0.004) followed with significant but lower positive effect sizes. Alternatively, in the second model, in which we replaced the actual number of bird species with the respondents’ estimation, the direction of the relationship reversed to a positive effect, and only the estimation of bird species’ number is a significant predictor of soundscape (p = 0.039). The water score was only marginally significant (p = 0.052). The second model performed better according to AIC values (2807.002 versus 2833.303).

Regarding safety perception, in model 1 the presence of signs of vandalism had a strong negative effect on people's perception of safety (p = 0.000), followed by a smaller positive effect of proportion of green within a 1 km radius (p = 0.004). We proceeded by adding to the model the control variables sex and income (significantly associated with safety in the SEM model), and the objective indicator of criminality. In this second model, which performed better (AIC 3029.772 versus 3188.77), vandalism kept the strongest effect on safety perception (p = 0.000), followed by the participant’s sex, with being female having a smaller and negative effect (p = 0.000). Proportion of green within 1 km radius lost relevancy, as well as income.

## Park qualities effect on wildlife support

The lowest number of observed bird species in the reference samples from the eBird dataset was found at Independência park, with 7 species, and the highest number was observed in Nove de Julho park, with 64 species. The mean urbanity index was 0.85 (in a range of 0.58–1.0). Effort time (minutes of observation) did not have an effect on the outcome variables, and the models that included it as a covariate resulted in higher AIC values. Therefore, only the best models (without this covariate) are reported.

**Figure 5:**
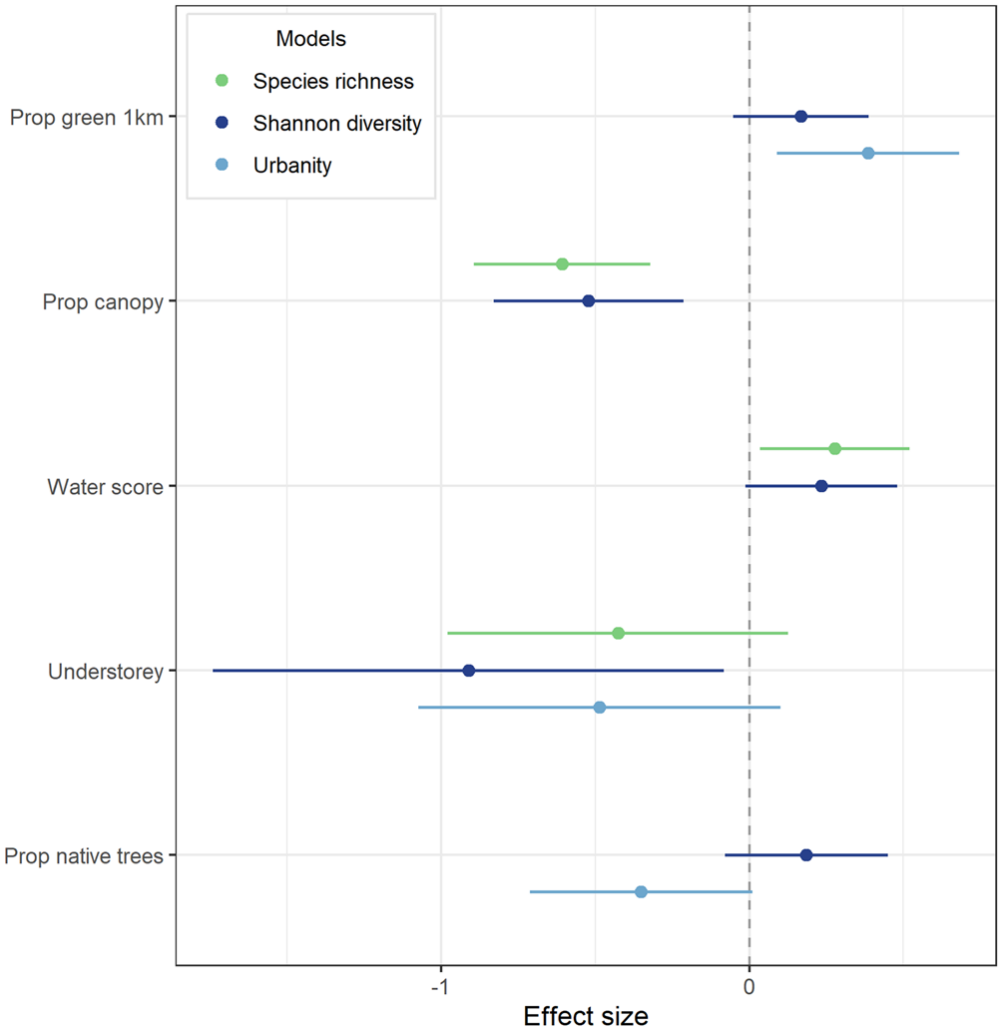
Standardized coefficients and 95% confidence interval of each variable included in the final models of bird community support (species richness, diversity, and urbanity). Variables with confidence intervals (bars) that do not cross 0 are statistically significant. See Table 2 for definition of variables.

**Figure 6:**
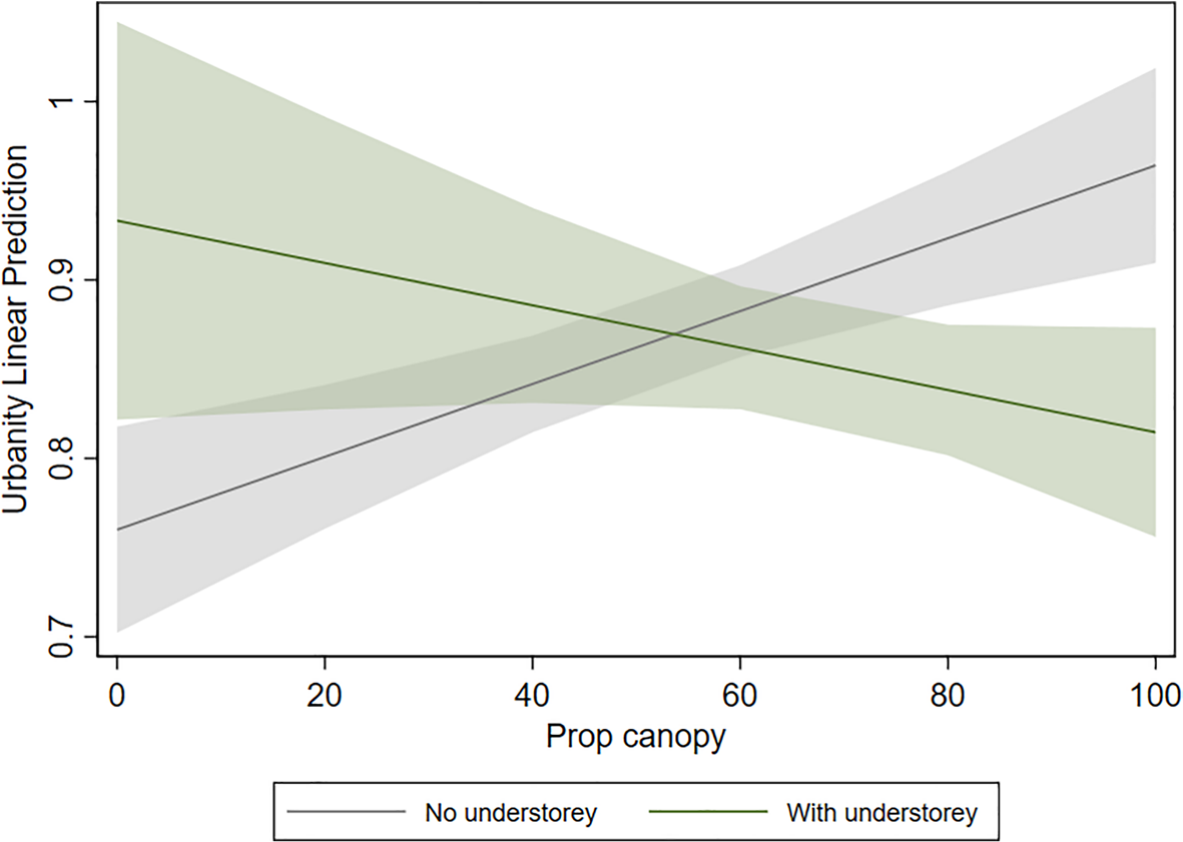
Relationship between proportion of tree canopy (Prop canopy) and urbanity index in the presence or absence of understory vegetation. Shaded area represents the 95% confidence interval.

Higher estimates of bird species richness are associated with a lower proportion of tree canopy (p = 0.000) and higher water score (p = 0.036) (Fig. 5). Shannon diversity is negatively affected by proportion of canopy (p = 0.003) and the presence of understorey vegetation (p = 0.043). A high urbanity index is associated with a high proportion of vegetation within 1 km (p = 0.018). The interaction effect between proportion of tree canopy and presence of understorey was significant only in the urbanity index model, showing a negative relationship with urbanity (p = 0.002). Sites with 80–100% of tree canopy and with understorey vegetation are predicted to show a significantly lower urbanity index than sites with the same canopy proportion but without understorey (p = 0.003 and p = 0.001 respectively, Fig. 6).

A summary of the results of setting perceptions and wildlife support models is available as Supplementary Material (Supplementary Fig. 4). This figure highlights the factors that were significant in both objective and subjective models of setting perceptions, and allow the identification of synergies and trade-offs between the human and animal dimensions.

## Discussion

### Park quality and user restoration

Our findings reveal that urban parks can show significant variation in their restorative potential, and may in some cases not even differ to a busy city square. The quality of these spaces plays a decisive role in the provision of psychological restoration opportunities linked to improved well-being.

Safety perception was the strongest factor affecting the perceived restorativeness of park users. Feeling safe in the environment is a basic condition to permit the rest of directed attention^28^ and this finding reflects that vulnerability to criminal acts in such open spaces may hinder green space benefits. Naturalness perception—the similarity with a natural and biodiverse environment—had the second strongest effect on perceived restorativeness. Nature fulfills two main components of the restorative experience: “fascination” towards living beings and processes, and “being away” to a setting with a different content than everyday life^28^. Provided safety conditions, high naturalness perception is the main factor to be aimed for in park design for improved restorative potential.

Vegetation coverage within and adjacent to park boundaries indirectly affected users’ perceived restorativeness through their perceptions of naturalness and soundscape. The beneficial effect of higher levels of tree canopy cover can be associated with the effectiveness of tree belts and street trees as barriers to environmental noise, and even beyond, vegetation may also induce a greater perception of noise reduction than the actual values^29,30^. A park with high tree canopy coverage may evoke familiarity with the original environmental condition as São Paulo is embedded in the Atlantic Forest biome, in which mature forests are made-up of big trees, an abundance of epiphytes, and multiple vegetation layers^31^. This forest density is also a potential explanation for the positive effect of understorey vegetation on naturalness and management perception, suggesting that it did not create a perception of overgrowth and unmanaged vegetation but rather an intentional decision to foster the natural aspect of the vegetation patch. Contrary to our expectations based on previous studies^32,33^, perceived safety was not associated with park configuration or vegetation aspects. This means that management actions such as the removal of understorey vegetation for improved visual permeability^32^, in this case, actually weaken restorative outcomes and do not necessarily improve the user’s sense of safety in the park.

Additionally, aspects of vegetation composition affected the way people perceived park management. Higher perceptions of management were associated with a higher number of tree species (objective and perceived) but in a lower proportion of native in comparison to exotic species. This finding can be associated with previous studies showing that park users are more attentive to ornamental and emblematic species, which are usually exotic species intentionally cultivated by gardeners^34^. This effect is more likely associated with their attractiveness than with the identification of their “nativeness” by the general public, as non-native species may be seen as unusual, more colorful, and interesting^35^. Therefore, park users correctly associated the increasing presence of ornamental/exotic trees with higher levels of intervention and vegetation care.

The presence of water features is known to positively affect nature^36^ and soundscape perceptions^37^, and the degree to which water benefits restoration depends not only on its presence but also its accessibility^38^. In our study, a combination of accessibility (possibility of visual, acoustic, and/or physical contact) and the natural condition of the water body yielded the best outcomes for restoration through greater naturalness and soundscape perceptions. This aspect is highly relevant in a context where water bodies are often present in parks but, due to their poor ecological condition, are isolated or hidden from users through physical barriers or a design that does not encourage contact with this feature. In this sense, efforts in the ecological restoration of water bodies within parks are highly recommended.

An aspect of site management is of high relevancy for parks’ restoration potential. Visible signs of vandalism in park facilities have the greatest effect on management and safety perceptions. The effect on management perception is related to the evidence of the inability of the administration to repair or replace damaged facilities. The presence of clear signs of vandalism in the park such as graffiti and broken facilities are the main predictors of safety perception whereas a quantitative indicator of criminality in the region is not relevant, which can be explained by the “Broken Windows Theory”^39^. The theory states that when signs of disorder are left unrepaired the feeling of carelessness raises fearfulness in residents, which may not be associated with an actual increase in crime rates. Our findings also suggest that park quality may reflect neighborhood socioeconomic level, as respondents’ income is not associated with perceived safety when accounting for park characteristics. In this regard, focusing financial resources on parks’ maintenance would be an effective measure to improve safety perception and consequently boost their restorative potential.

An additional important factor for safety perception is sex disparity. Even when accounting for park quality and other control factors, sex remained a relevant factor with being female positively associated with unsafety perception, similar to findings in urban green spaces of other Global South countries^32^. The lower share of female respondents in our sample compared to the general population of São Paulo suggests that females might be underrepresented in these spaces in a reflection of safety issues. Considering that females are at higher risk of developing mental health issues than males, and may benefit more from urban green spaces^40^, it is essential that public policies promoting the use of open (green) spaces also focus on female's safety, ensuring that they have equal opportunity to enjoy the whole potential of urban parks for their health benefits.

### Park quality and wildlife support

Regarding the wildlife dimension, not only the presence but also the naturalness of parks’ water bodies matters for bird species support in terms of overall richness. Microclimate effects such as milder temperatures close to water elements, as well as the habitat and food resources provided by natural water bodies, were proposed as possible reasons for the positive relationship between water and bird richness already reported in São Paulo city^41^. On the other hand, the negative effect of higher levels of closed canopy on bird species richness and diversity contradicts previous studies in urban areas^42^ but is in line with other findings^43^. The positive effects of water presence and lower proportion of tree canopy suggest that bird richness in these urban parks is driven by habitat heterogeneity. Green spaces that provide multiple microhabitats such as forest patches, open vegetation, and water bodies, offer a broader range of resources and can attract bird species with varying habitat and foraging requirements^43^. The association between understorey vegetation and bird diversity may be explained by an unbalanced presence of forest-dependent species in parks with forest remnants and larger forest patches. Notably, these results were influenced by the high diversity estimates in parks located at wetlands on the border of Guarapiranga water reservoir, which present the lowest canopy proportions in our sample (around 20%) but are habitats of good ecological quality. This also indicates that tree canopy cover alone may not be used as a proxy of ecological quality for the bird assemblage, at least in the environmental configuration of São Paulo city’s parks.

When looking at a qualitative aspect of the bird assemblages in each park, the interaction between vegetation aspects was relevant and resulted in distinct outcomes. While higher canopy proportion and the presence of understorey negatively affected diversity estimates, the combination of these aspects reduced urbanity levels of the bird assemblage. This means that bird species more sensitive to human disturbances—and of higher conservation value—are benefitting from higher park tree canopy proportion when accompanied by understorey vegetation. The presence of understorey vegetation improves the ecological quality of urban forest patches in terms of providing nesting sites, increased forage availability, protection against predators and domestic animals, and is especially important to small-body and ground/understorey-nesting species, which are more sensitive to anthropogenic disturbance^44,45^.

It is important to emphasize that this analysis did not aim to investigate all relevant features driving bird diversity estimates in urban parks, therefore our results must be interpreted in the context of comparison with the human dimension. Several green space features that are known to affect urban wildlife were not considered due to their irrelevancy for psychological restoration outcomes. Additionally, the analysis could not take into account green spaces with very low ecological quality due to either the lack of bird checklists or insufficient sampling effort in such areas. Bird watchers are usually attracted to more natural-like areas where the potential for bird encounters is higher. The inclusion of bird surveys in parks of lower ecological quality would contribute to the clarification of the tree canopy effect, especially in sites with lower than 40% of tree coverage.

### Parks for humans and wildlife

Park users’ impression of being in a place similar to a natural habitat with several animal and plant species (naturalness perception) was the main setting perception contributing to restorativeness, being more relevant than the perception of maintenance and facilities. This finding suggests that the design and management of parks that maximize the restorative potential should focus on promoting nature rather than overly manicured green spaces. This is a synergy with what is also necessary to effectively provide habitats for different wildlife species and to promote the environmental quality of the green space itself.

We identified synergies and trade-offs between significant green space indicators for users’ mental restoration and bird assemblage support. A clear synergy is the presence of water bodies, especially with a natural aspect, which improved bird richness as well as restoration through higher perceptions of naturalness and soundscape. Therefore, this is a feature that should receive high relevance in the design and management of green spaces.

On the other hand, proportion of canopy cover presented both synergies and trade-offs depending on quantitative or qualitative aspects of bird species support. While higher tree canopy proportions with understorey vegetation provide higher restorative potential to users and benefit bird species of higher conservation value, it also reduces bird diversity estimates. However, birds were the only animal group considered in this study, and it is likely that other groups (from larger mammals to arthropods) do respond differently to canopy proportions and understorey vegetation features. Although the positive effect of lower tree canopy on diversity estimates may be a trade-off with psychological restoration, it could be considered a synergy with other human benefits, as a park with different habitats may provide more opportunities for recreational activities.

Although the proportion of native tree species was not significant in the wildlife models (only marginally significant for the urbanity index), the negative relationship of higher proportion of native tree species and management perception of users can be discussed in the light of existing evidence. Studies conducted in Brazil^46^ and abroad^47^ have shown that exotic tree species can provide important resources across seasons for birds in urban areas. Although the presence of exotic tree species in urban parks might be seen as a synergy between restoration and support to birds, the mega biodiversity of Brazil must stimulate the exploration of native plant species^46^ that are less known or still not used in urban areas. Choosing native plant species which can provide the attractiveness and resources that the exotics are currently offering to humans and animals and which are naturally adapted to local conditions would boost biodiversity conservation and resilience in cities.

Our results contribute to the emerging evidence of the beneficial effect of biodiversity on human health and well-being^19,20, 48^, through the psychological restoration pathway. Regardless of the actual number of bird and tree species, people´s estimation of biodiversity was of high relevance to the three domains of setting perceptions related to restoration potential. Higher estimation of bird species by users was associated with better soundscape perception as the single predictor in the best model, in opposition to actual bird richness. The mismatch between people's perception of biodiversity and objective indicators was reported before^34^ and the general underestimation of bird richness in our case may be due to either short visit duration that does not allow the encounter with many species, the lack of knowledge on local biodiversity, and due to the cumulative aspect of the bird inventories (See Methods section). In this sense, investment in environmental education of the population on local biodiversity could lead to enhanced benefits to mental well-being. As urban parks are the logical places for connecting with nature in urban areas, simple interventions such as guided tours and biodiversity-focused signage can improve visit quality and hence boost the restorative experience. Furthermore, future studies could explore and validate some of the associations identified here using qualitative data such as interviews with park visitors, as well as improve the wildlife data carrying out bird surveys especially on the same days as questionnaire application.

## Conclusions

This is the first study to provide quantitative evidence on the restorative potential of urban parks in Brazil, contributing to the understanding of pathways linking the environment to human health benefits. The findings highlight that urban parks can play an important role in mitigating the urban psychological penalty, or the negative effects of city on mental health and well-being, offering opportunities to recover from depleted psychological capacities and stress. To maximize this potential, safety perception should be prioritized as a fundamental condition for restoration in São Paulo’s urban parks, while design and management should focus on features that enhance the naturalness perception of users, the second key factor contributing to perceived restorativeness of users.

We show that park quality is crucial in determining its restorative potential for visitors. Settings of high restorative potential should have a forest-like appearance with abundant tree canopy (> 60%) and multiple vegetation layers, and feature numerous tree species (including exotic ones). Additionally, the presence of easily accessible natural water bodies can enhance the restorative experience further. In terms of management, financial resources should be prioritized for the repair of signs of disorder such as graffiti and broken facilities, avoiding the depreciation of park benefits due to safety concerns.

Interdisciplinary socio-ecological studies are important to shed light on potential synergies and trade-offs between different beneficiaries of green spaces. Through a holistic approach, we were able to provide recommendations for the design and management of urban parks considering benefits to both human well-being and urban wildlife conservation. Taken altogether, our results suggest that incorporating natural water bodies into park design maximizes benefits for both dimensions. “Forest-like” parks provide higher restorative potential to users while benefiting bird species of higher conservation value. On the other hand, more heterogeneous parks may provide better bird diversity estimates, and, despite lower restorative outcomes, they could provide more diverse recreational opportunities to users.

Besides the intrinsic value of nature, the evidence that higher biodiversity level is also a relevant factor influencing human well-being reinforces the importance of investing in urban nature. We underscore that attention to green space quality is essential to effectively provide the expected benefits for human health and biodiversity conservation. Our findings support efforts towards healthier and biodiverse cities through the provision of a heterogeneous green infrastructure consisting of a network of spaces that are purposely designed and managed to reconcile both human and wildlife needs.

## Methods

### Study area

**Figure 7:**
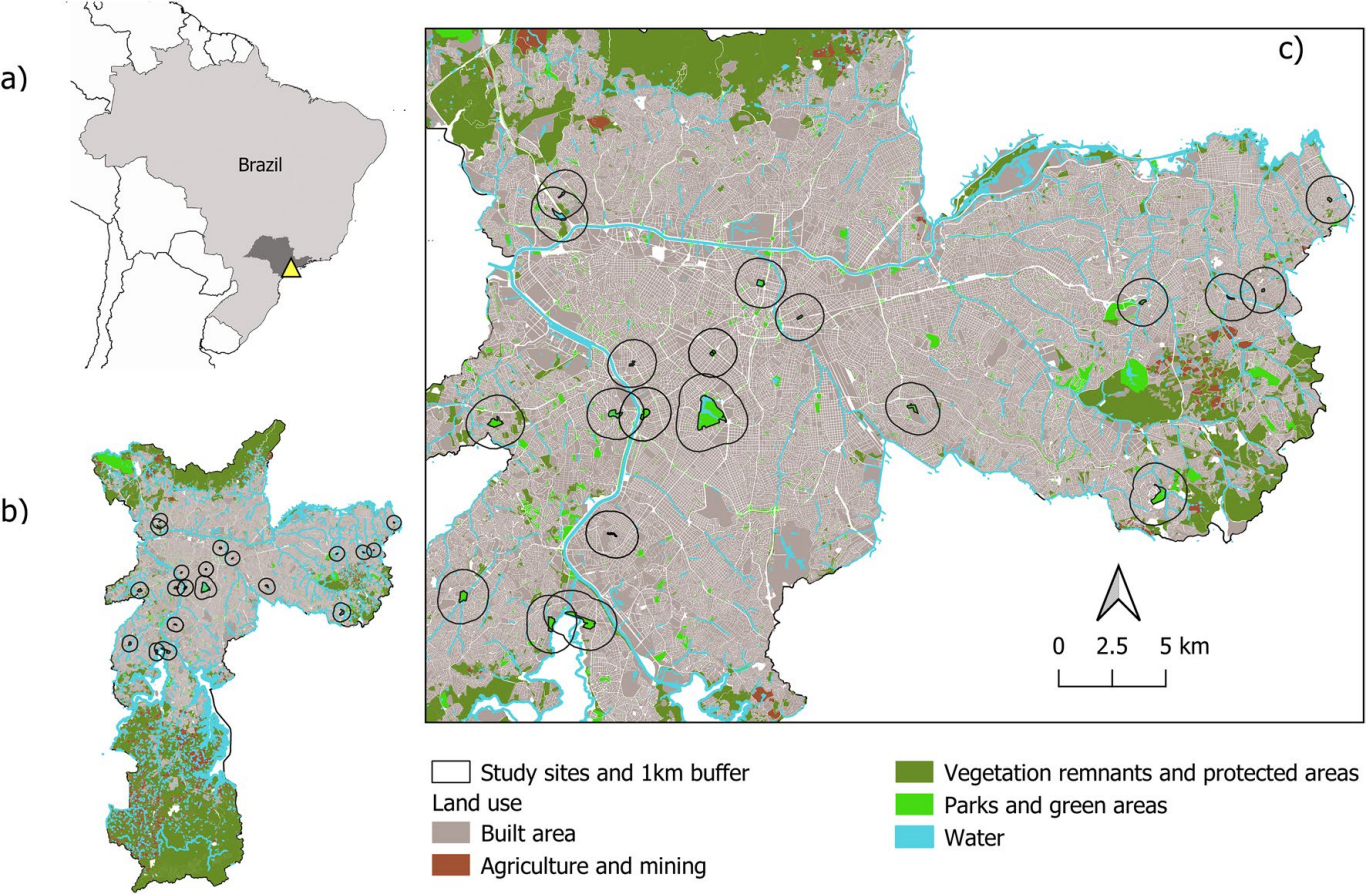
Maps showing the (**a**) Location of the city of São Paulo (yellow triangle) within the state of São Paulo (dark gray) and Brazil. (**b**) Land use map of São Paulo municipality showing the distribution of vegetation and green areas in relation to the build area, with study areas and their 1 km buffers in detail (**c**). Land use data downloaded from GeoSampa platform (https://geosampa.prefeitura.sp.gov.br/).

The city of São Paulo, located in southeastern Brazil (Fig. 7a), is the fourth largest urban agglomeration (22 million inhabitants)^49^, and has one of the highest prevalence of mental disorders in the world (29.6%), with 10% of severe cases^50^. This megacity overlaps the Atlantic Forest biodiversity hotspot^51^ and 48% of its territory is covered by vegetation (of different types). Despite the high share of vegetation, it is unequally distributed across regions (Fig. 7b), with coverage ranging from 16% in the center to 62% in the southern region^52^.

The existing 111 municipal parks are categorized as urban, linear, reserve, and natural. Reserves and natural parks are intended for biodiversity conservation, and because their accessibility for the public is restricted, these categories were not considered in this study. According to the municipality, urban parks are structured fenced spaces that protect biodiversity and provide recreational and sports facilities, whereas linear parks are riverside areas usually not fenced. The latter may or may not present recreational facilities as their main objectives are the protection of areas adjacent to water bodies and the connectivity between green areas. Looking at the characteristics of these spaces, there is a broad range of variation in aspects of the spatial configuration, vegetation structure, design, and management, making them a useful case to test the effect of park quality on our response variables.

### Research design

An observational study with cross-sectional data collection was conducted capturing ecological and social perspectives in the context of urban green spaces. Primary data was collected through a questionnaire elaborated to assess park users’ perceptions and socio-demographic aspects. Secondary data was retrieved to compute green space indicators and biodiversity variables.

Our analytical approach comprised three main steps (Fig. 1). First, we investigated the effect of users’ perceptions of the setting on restorativeness by conducting a structural equation model, as this analysis considers the particularities and errors attached to self-reported data and the use of psychometric and rating scales. In the second step, we used mixed models to assess associations between objective indicators of green space quality and users’ perceptions of the setting. Finally, through linear regressions, we assessed the effect of green space indicators on wildlife support variables.

### Wildlife data

Urban wildlife support in this study is represented by the bird assemblages observed in each park. Birds have been used as biodiversity indicators for several reasons, such as relatively easy identification and data availability, and are conservation flagships due to the interest and concern by the public^53^. We used two different sets of secondary data to obtain metrics for human perceptions and wildlife models.

The number of bird species observed in each park was used as an independent variable in the human perceptions models. Data were extracted from the Wildlife Inventory of São Paulo city^54^, which present a cumulative species list for each park, including observations since 1993. Surveys comprise transect counts, recording of birdsongs, and mist-net sampling. More recently, these lists also include observations collected in a structured citizen science program conducted by the municipality to promote bird watching in parks (i.e., “Vem Passarinhar”).

For the bird support models, we opted to use data from the citizen science platform eBird (https://ebird.org), which provides checklists including the abundance of each observed bird species, thus allowing the estimation of standardized bird species diversity estimates. eBird is a data source of bird observations from a global network of volunteers, which follow collection protocols and data quality checks through automated filters and experts review^55^. Checklists from São Paulo state were obtained within the timeframe of January 2009 to July 2022.

### Selection of parks for survey

To enhance the representativeness of our set of study sites considering the variation found in city parks’ characteristics, we adopted a stratified sampling approach to select sites from the total of 92 urban parks under the ownership of the municipal government that were legally created and qualified until 2019, and accessible to the general public. As 90% of these parks are below 20 ha in size, we set a cut point value of 10 ha to categorize them into smaller or larger parks. Six groups were created based on park area and visual estimation of tree canopy cover (low, medium, or high). Three parks from each group were selected representing, as much as possible, different levels of socioeconomic vulnerability (low, medium, or high) classified within a 1 km radius of park boundaries (Fig. 7c). Besides the selected 18 parks, the most popular city park (Ibirapuera) and a busy central square (Largo da Batata) were included representing best and worst cases (see Fig. 2 for a list of study sites). For this preparatory assessment, we obtained geo-referenced data on park boundaries and the Paulista Index of Social Vulnerability at the census tract level from the GeoSampa platform kept by the Municipality of São Paulo (geosampa.prefeitura.sp.gov.br) and combined them with Google Satellite images on QGIS 3.10.5.

### Questionnaire with park users

An on-site questionnaire survey with visitors of each study site was conducted from March to June 2019, during the dry season. Each site was visited twice, on a workday and a weekend day, from 8 am to dusk, and under similar weather conditions. Respondents were randomly approached among people that were visiting the park. Eligible participants were Brazilian adult (≥ 18 years old) residents of the city. Participation was anonymous and subjected to the signature of an informed consent form by all subjects and/or their legal guardian(s). The questionnaire was designed in a sequence aimed to reduce response bias and was applied by trained interviewers. Cards with response options for the questions were offered to avoid answer errors. The average time to complete the questionnaire was 15 min. This study was approved by the Brazilian National Committee on Research Ethics (CONEP, CAAE: 00239018.7.0000.5390), and all procedures complied with ethical guidelines on research with humans.

The questionnaire was divided into four sections: self-reported health and stress level, perceived restorativeness, setting and biodiversity perceptions, and sociodemographic factors. The first section comprised the individual health perception made up of three items (general health, mental health, and well-being) evaluated on a 5-point scale. In addition, stress perception in the last month was measured by the Perceived Stress Scale^56^, which is composed of 10 items evaluated on a 5-point scale validated to Brazilian Portuguese^57^. The second section included a version of the Perceived Restorativeness Scale^58^ composed of 15 items evaluated on a 7-point scale, from 0 to 6, comprising the being away, fascination, and compatibility dimensions, and validated for the target population^59^. The third section comprised a scale to assess perceptions of the setting in three dimensions (3 items each, 7-point scale) built upon previous studies^60,61^: soundscape perception on pleasantness, eventfulness, quietness (e.g. “This park has a pleasant soundscape”); management perception regarding maintenance of facilities and vegetation, and overall cleanliness (e.g. “This park’s vegetation is well cared for”); naturalness perception in terms of similarity to nature and biodiversity (e.g. “This park looks like an untouched nature”). Additionally, single questions were used to measure the perception of safety (i.e. “This setting transmits a sense of safety”, 7-point scale), and biodiversity estimation (i.e. “About how many species of birds and trees would you say exist in this park?”). The response to the latter question consisted of five intervals of species estimation for trees (up to 50, 51–100, 101–150, 151–200, more than 200) and birds (up to 15, 16–30, 31–60, 61–100, more than 100) according to the range found in the parks’ inventories. The fourth section included socio-demographic self-reported questions such as sex, age, and income.

### Park quality indicators

Data on 38 indicators reflecting park quality were collected for the sampled sites, comprising aspects of spatial configuration (e.g. proportion of tree canopy coverage), vegetation structure (e.g. proportion of native tree species), design (e.g. number of habitats), management (e.g. cleanliness), and adjacent landscape (e.g. vegetated area within 1 km radius). Indicators were selected from a previous study that explored green space characteristics affecting wildlife support and mental health^12^ and in participatory consultation with practitioners from the São Paulo municipality through a workshop conducted in 2019. This consultation aimed at tailoring the analysis towards results that could be easily translated into practical recommendations on the planning and management of green spaces. A first filter was applied using a Pearson correlation matrix to identify and exclude indicators that were highly correlated (r > 0.80) with two or more indicators, leaving a total of 19 to be considered in the following analyzes (Supplementary Table 3).

Data on vegetation coverage was obtained from the Digital Mapping of São Paulo Vegetation Cover^52^. Vegetation patches were identified and classified into 15 categories on a 1:1.000 scale, based on orthophotos of the year 2017/2018 with 0.12 m of resolution and 3D digital mapping (LiDAR). Plant diversity metrics were calculated based on the Flora Inventories of Municipal Parks^62^. Indicators of park management and design (Table 2) were collected mainly on-site on the same days of the questionnaires’ application.

**Table 2:** List of green space indicators used in the final models of setting perceptions. For continuous variables, the mean number or percentage across parks and range is provided. For categorical variables (defined as 0 or 1), the frequency refers to the percentage of parks in the mentioned category.

Variable	Mean (range)/frequency	Definition
Landscape		
Landuse	68% mixed	Dominant land use type surrounding the park: residential—0, mixed (residential and commercial or industrial)—1
Prop green 1 km	21.9% (8.8–47)	Percentage of area covered by vegetation within the total 1km buffer area surrounding the park
Spatial configuration		
Area	162,315.6 m^2^ (14,164–1,241,740)	Total park area
PA ratio	0.023 (0.005–0.049)	(Perimeter-area ratio) perimeter divided by total area
Prop canopy	61.9% (0–99.7)	Proportion of park area covered with closed tree canopy (trees’ crowns predominantly touch each other)
Prop open veg	20.2% (0–76)	Proportion of park area covered with herbaceous strata with or without sparse trees
Vegetation structure		
Tree species	112.6 (40–329)	Total number of tree species
Tree sp/ha	18.0 (1.72–49.42)	Number of tree species divided by park area (in ha)
Prop native trees	52.9% (27.8–84.9)	Proportion of species categorized as native out of the total richness
Prop native bushes	52.6%(0–100)	Proportion of bushes categorized as native out of the total richness
Design		
Water score*	1.21 (0–3)	Multiplies the presence of water body (no-0/yes-1) and its accessibility (no-0/yes-1), plus water body naturalness (artificial-0/natural-1)
Number habitats	2.2 (1–4)	Number of microhabitats within the park
Topography	79% flat	Predominant topography: Flat or slightly undulating—0, uneven—1
Management		
Understorey	58% no	Presence of vegetation layer(s) beneath the tree canopy: no—0, yes—1
Cleanliness	58% with trash	Reflect the presence of trash in the days of survey: clean—0, with trash—1
Vandalism	74% no vandalism	Presence of signs of vandalism in park facilities: No vandalism—0, vandalism—1
Wildlife		
Bird species	69.4 (17–223)	Total number of bird species listed in each park

*For wildlife models, water score did not consider accessibility.

### Data analysis—factors affecting restorativeness

We tested construct validity of the psychometric scales through reliability and factorial validity analysis. We adopted recommended thresholds indicating adequate parameter values and good model fit as Cronbach’s alpha (α) higher than 0.70^63^, Comparative Fit Index (CFI) higher than 0.95, Root Mean Square Error of Approximation (RMSEA) lower than 0.06, and Standardized Root Mean Square Residual (SRMR) equal to or below 0.08^64^. These analyses were performed in IBM SPSS Statistics 24 and R software version 4.0.0 using the ‘lavaan’ package (version 0.6-12).

For the Perceived Stress Scale and Perceived Restorativeness Scale we conducted confirmatory factor analyses (CFA) since their structures were analyzed before^57,59^. Both scales confirmed their one-factor and second-order structures with adequate internal consistency and fit indices (α = 0.81 and 0.92, respectively, Supplementary Table 4). The factorial structure of the Perceived Restorativeness Scale enables the calculation of a perceived restorativeness score (PRS score) based on the average of items from the being away, fascination, and compatibility dimensions. We then tested the sensibility of the PRS score to differences across sampled sites estimating margins of responses and 95% confidence intervals for each site and performing pairwise comparisons of the predicted margins. For this analysis, we used the software STATA 16.1.

The three items referring to self-reported health status were combined into a latent variable “Perceived health”, which presented adequate internal consistency (α = 0.76). For the settings perception scale, we conducted a Principal Components Analysis with three fixed factors, which confirmed that the items loaded in the proposed dimensions (soundscape, management, and naturalness), each of them with adequate internal consistency (α = 0.73, 0.88, 0.70, respectively, Supplementary Table 4). We then calculated an average score from the three items of each perception to be used as dependent variables in the regression models.

After confirming the robustness of our scales, we investigated how visitors' perceptions of safety and the setting, and personal factors affected restorativeness according to our theoretical framework (Fig. 1). Considering the nature of this subjective data, we conducted a structural equation model (SEM) with the latent variable perceived restorativeness (made up of its three dimensions) as the response variable, and the latent variables of settings perceptions (soundscape, management, and naturalness), as well as safety perception as predictors. In this analysis, we did not consider the park effect, but rather relationships at the individual level, and therefore we included the effect of control variables sex, age, income, and self-reported health (latent variables of health perception and stress perception). Income and safety perception were transformed into the dummy variables “up to (0)/more than four minimum wages (1)” and “safe (0)/unsafe (1)” (unsafe merged scores from 0 to 2).

The SEM analysis was conducted with the Weighted Least Squares Means and Variances (WLSMV) estimation method, which is more appropriate and with superior performance than Maximum Likelihood for the ordinal level of scale items and large sample sizes^65^. The model was subsequently optimized with the exclusion of non-relevant pathways. Indicators of good model fit followed the same thresholds mentioned for the psychometric tests. Analyzes were performed in R software using the ’lavaan’ package (version 0.6-12).

### Data analysis—park quality and user perceptions

To understand how park characteristics affected respondents’ perceptions of the setting, we first tested the necessity of multilevel analysis to account for the non-independency of observations (respondents clustered in parks) running null models (without predictors) including a random-effect (park) and checking a significant effect on intercepts and intraclass correlation^66^. As all models were significantly different from one-level linear models and intraclass correlation ranged from 0.158 to 0.367, we conducted mixed-effects linear models for each perception score (naturalness, soundscape, and management) as dependent variables, park indicators as fixed factors, and park name as random factor. Potential green space indicators for each perception were selected based on theory and evidence accumulated in the field (Supplementary Table 3). The number of bird species observed in each park was included as fixed effect in the models of naturalness and soundscape perception. Observations collected in the site ’Largo da Batata’ (N = 50) were excluded in all models, and observations in ’Benemérito José Bras’ (N = 56) were excluded in models of soundscape and naturalness, both due to incomplete biodiversity data.

As preliminary analyzes showed a poor correlation between the actual number of bird and tree species with respondents’ estimation of richness (see “Results” section), we contrasted models using only objective indicators (model 1) with models replacing objective indicators of species richness by respondent’s estimation of richness (model 2). For the species richness estimation variables, we used the mean number of species of the interval selected by the respondent in the questionnaire.

Additionally, we explored green space predictors of safety perception using a mixed-effects ordered logistic regression, considering that the response variable is a Likert-scale item^67^. We compared a model including only park predictors (model 1) with a model including control factors that could influence safety perception in the setting (model 2). These comprised significant personal factors resulting from the SEM analysis and an indicator of criminality level in the region (number of thefts registered in the closest police station to each park during the year 2018, source: Sou da Paz Analisa, soudapaz.org).

For all models, we checked multicollinearity between predictors and adopted a full model approach, constructing models that included all variables with acceptable variance inflation factor^68^ (VIF < 10) (Table 2). Models were run with robust estimation of standard errors of regression coefficients, which account for data heteroscedasticity and other violations of distributional assumptions^67^. Model diagnostics included the exclusion of influential values based on Cook’s distance and residual plots to check the normality and homoscedasticity of residuals. We compared model performance through the Akaike Information Criteria (AIC), with lower values indicating a better fit. We further explored statistically significant variables of the models calculating predictive margins for different levels of the response variables keeping all covariates constant. All analyzes were conducted in STATA 16.1. Visualization of models’ results in the form of coefficient plots (with standardization of continuous and ordinal variables using z-scores) was elaborated with the ‘dotwhisker’ package (version 0.7.4) in R.

### Data analysis—park quality and wildlife support

We constructed models of bird species support based on three different response variables calculated from the eBird dataset. We followed recommended steps for data clearing and filtering^69^ using only complete checklists, stationary or traveling protocols, and merging duplicate lists according to sampling event identifier. A dataset with checklists for each park was created intersecting geographic coordinates from the checklists with the shapefiles of park boundaries. A reference sample for each park was selected based on the following criteria: observation period ranging from 60 to 100 min, number of observed specimens available for all species reported (removal of lists in which bird species were reported as “X”—no count), and finally, the higher number of species reported. The definition of the range of observation period aimed to reduce variability in sampling effort while allowing enough time for observation of a high number of species^70^. After applying the criteria, the timeframe of the selected checklists was reduced to the period of 2016 to 2022. We used a sample of 30 parks according to data availability on park characteristics and compliance with sampling effort criteria. A matrix with individual counts for each species by park was used as input to estimate species richness and Shannon diversity index on the ‘iNEXT’ package (version 3.0.0) in R. Using the ‘estimateD’ function, these diversity estimates were computed by standardizing samples by coverage^71^, at the minimum sample coverage level among all sites. Additionally, we calculated the urbanity index of each park’s checklist. This index (adapted from the urbanophobe index^42^) considers the degree of vulnerability of species to anthropic disturbance, commonly found in urban environments. The level of sensibility of each bird species (low, medium, high) was assessed in available literature^72^ and the index is calculated as the share of species with low sensibility out of the total number of bird species. A high index reflects a bird assemblage composed of a majority of species more tolerable to human disturbances instead of species that require a higher level of habitat quality.

Firstly, we tested if our response variables were spatially correlated through the Moran’s I test using the package ‘ape’ (version 5.6-2) in R. As spatial auto-correlation was not significant for all outcomes variables, we proceeded to run three linear regression models including all predictors that were statistically significant in the analysis of setting perceptions. This is in concordance with the aim of the study of analyzing potential synergies and trade-offs between human and animal dimensions using the same indicators. As park area was not correlated with bird richness (r = 0.30, p = 0.103) and diversity (r = 0.28, p = 0.137), we did not include it as a control variable in the models. However, we tested concurrent models by adding the sampling effort time (duration of bird survey) as a covariate. Due to the limited sample size, we used ‘lasso’ command to select the best predictive variables to be included in the final models. We adopted model selection and diagnostic approaches already mentioned in Sect. 3.5.3. Apart from investigating the main effects of variables, we ran additional regressions testing an interaction effect between proportion of canopy and the presence of understorey on all three independent variables. All analyses were conducted in STATA 16.1 and the coefficient plot in R.

### Supplementary Information

Supplementary Figures.

Supplementary Tables.

## Data Availability

The data generated for the current study are not publicly available due to further analyses being planned, but de-identified data may be made available from the corresponding author on reasonable request. For queries about the specific data and analysis, including r script, used in the present manuscript please contact the corresponding author.
