# Supplementary material for: Urban park qualities driving visitors mental well-being and wildlife conservation in a Neotropical megacity
Source: Sci Rep. 2024 Feb 28;14:4856. doi: 10.1038/s41598-024-55357-2 (PMC10902329; doi:10.1038/s41598-024-55357-2)
Supplement: Supplementary file 1 — Supplementary Figures. [file 41598_2024_55357_MOESM1_ESM.pdf]

Urban park qualities driving visitors mental well-being and wildlife conservation in a Neotropical megacity

Jéssica Francine Felappi, Jan Henning Sommer, Timo Falkenberg, Wiltrud Terlau, Theo Kötter

Supplementary Information

Supplementary Figure 1. Adjusted predictions of soundscape perception scores for each park and 95% confidence intervals. The dotted line represents the mean soundscape perception score of the total sample.

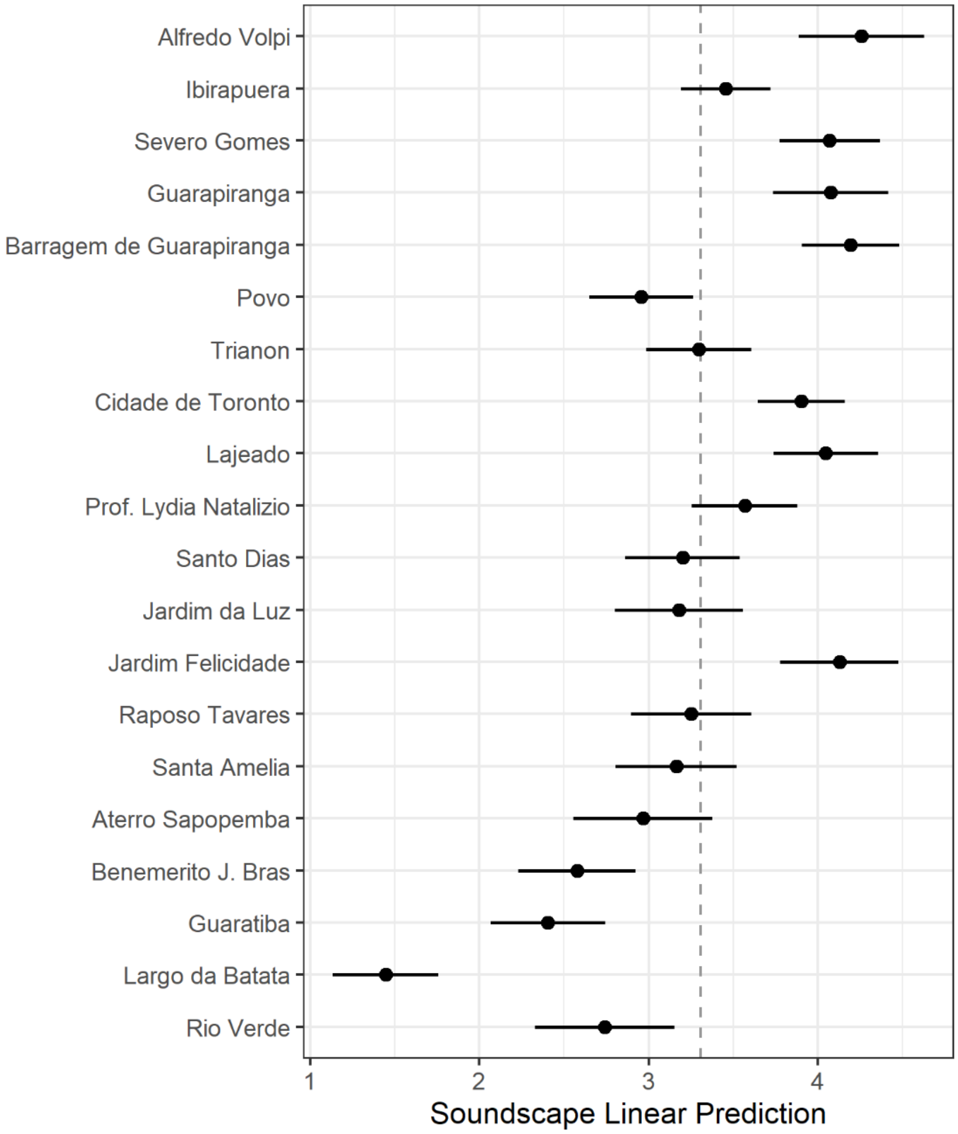

Supplementary Figure 2. Adjusted predictions of management perception scores for each park and 95% confidence intervals. The dotted line represents the mean management perception score of the total sample.

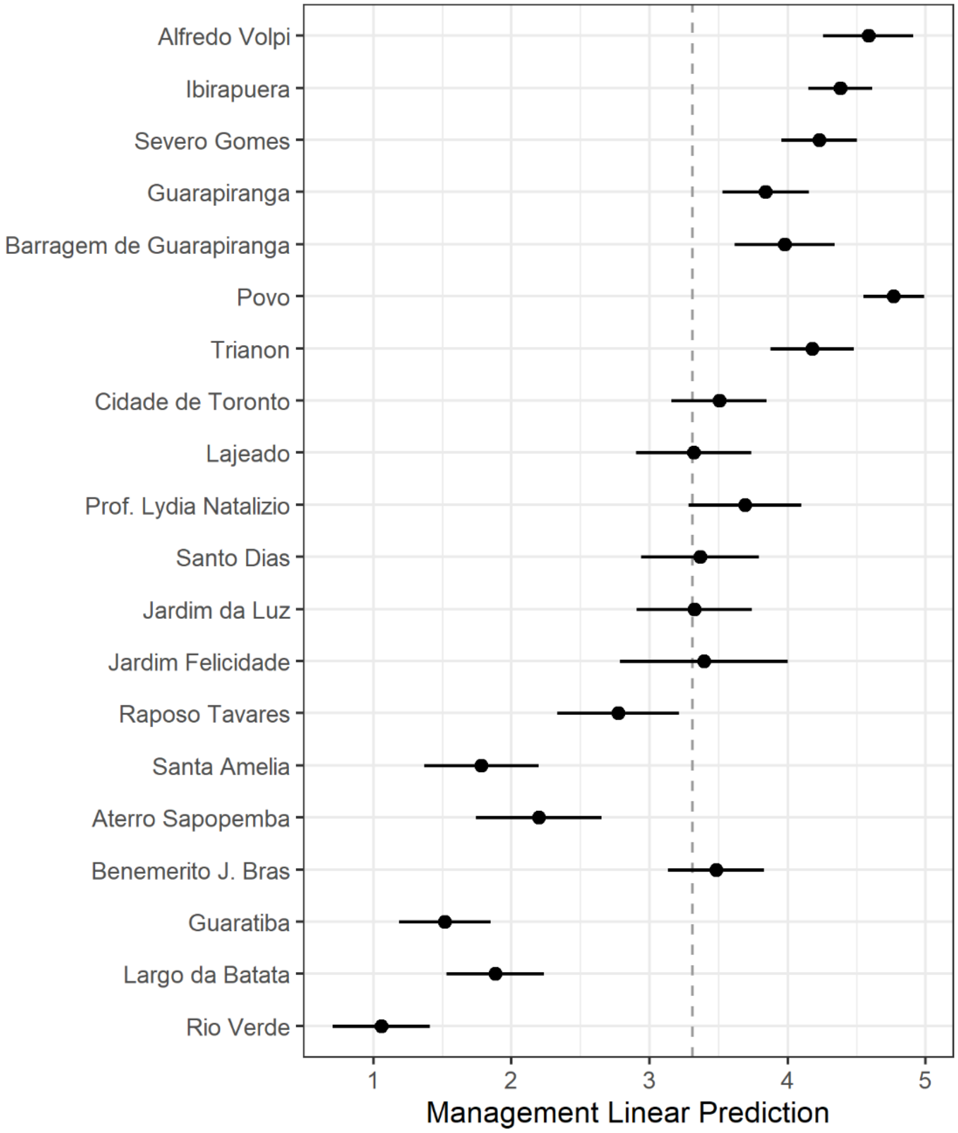

Supplementary Figure 3. Adjusted predictions of naturalness perception scores for each park and 95% confidence intervals. The dotted line represents the mean naturalness perception score of the total sample.

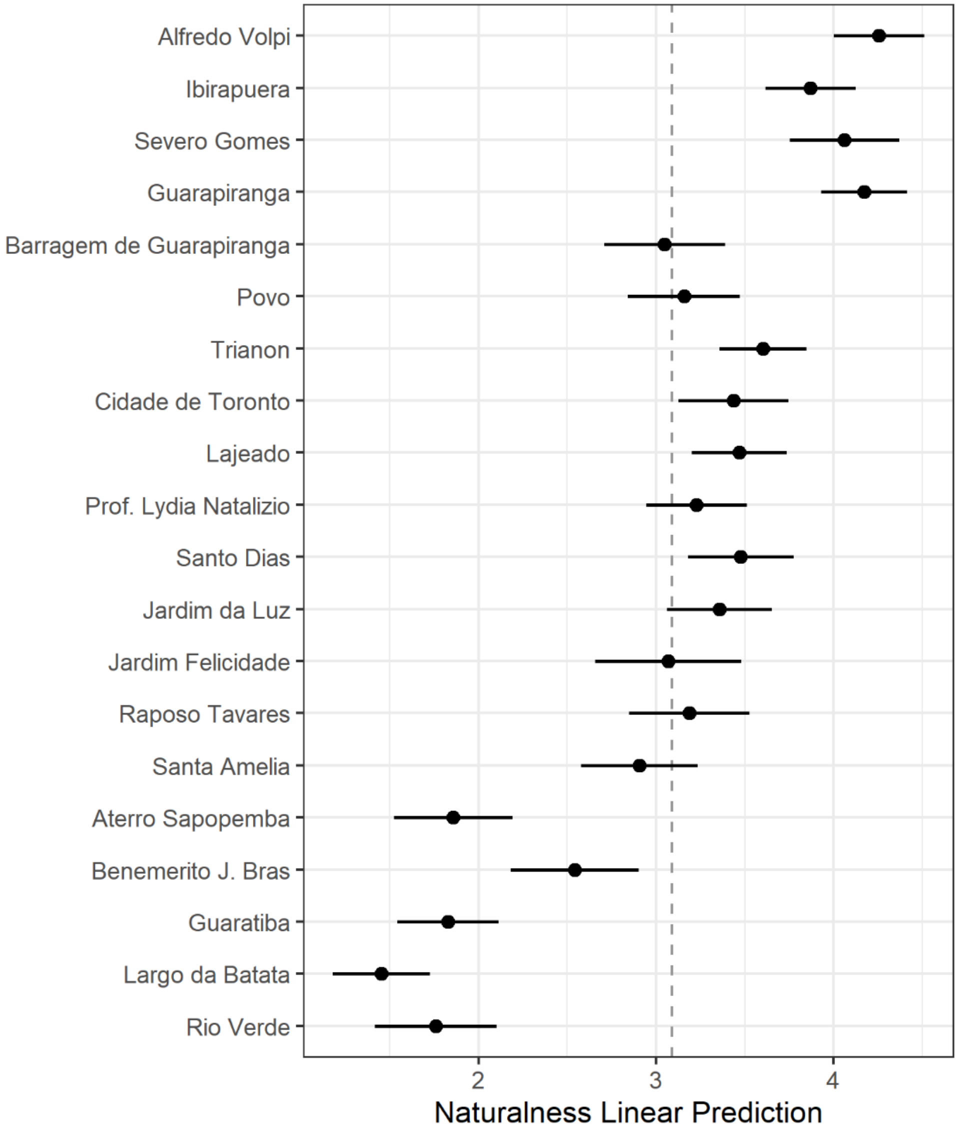

Supplementary Figure 4. Summary of the relationships found in models with perceptions and bird support metrics as dependent variables. Only statistically significant indicators are shown with the correspondent direction of the effect: positive (up arrow) or negative (down arrow) relationship. For the four perceptions, dark grey circles correspond to statistically significant indicators only in model 1 (objective indicators), hatched circles to statistically significant indicators only in model 2 (subjective indicators), and black circles indicators that were significant in both models. For the three wildlife metrics, dark grey correspond to the results of the main models and the hatched circle correspond to the single significant interaction model.

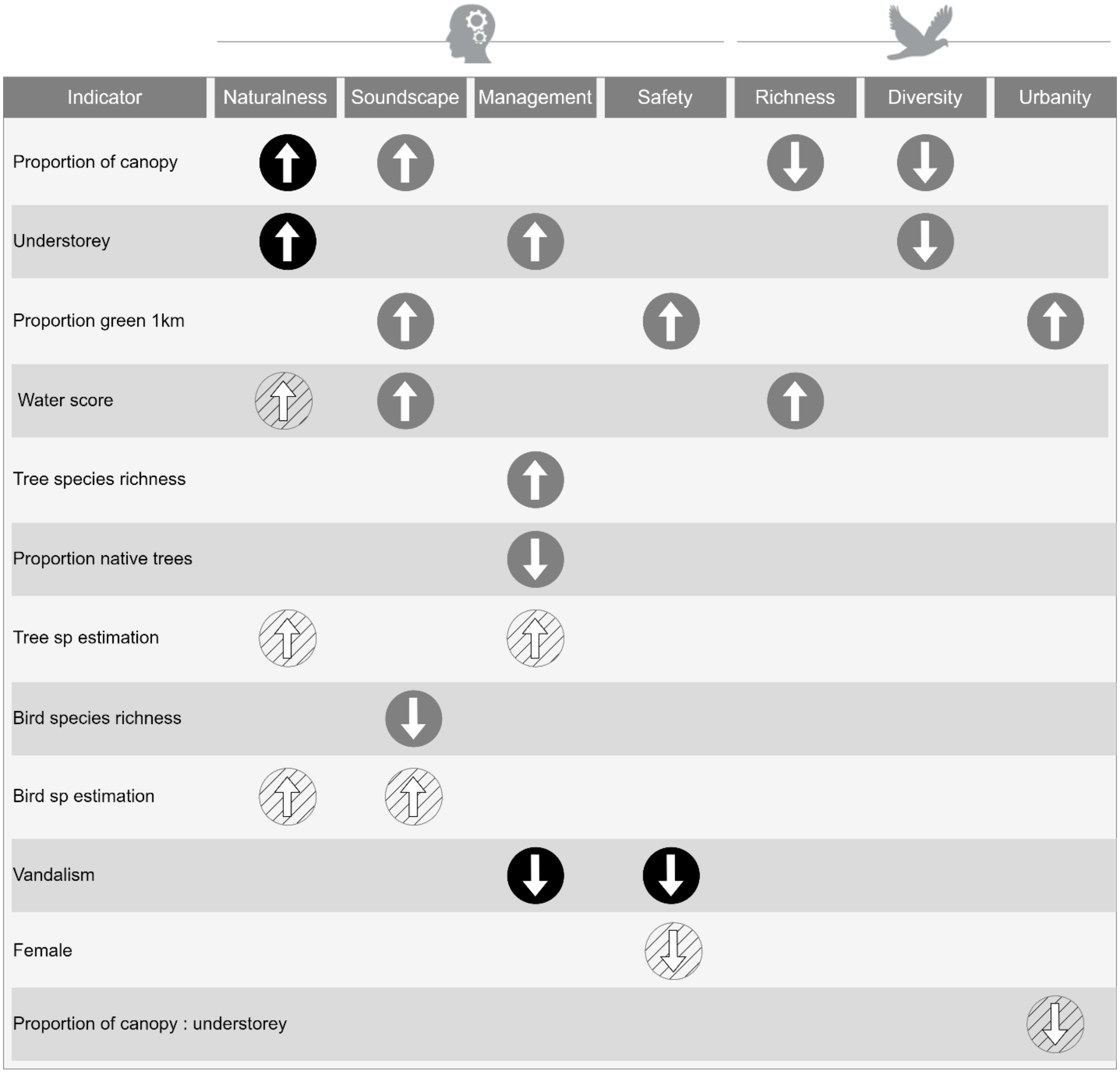

Table 1. Fit indices of the structural equation models.

Model	χ^2	df	CFI	RMSEA	SRMR
1. Full model (Perceptions + controls)	3176.218	755*	.935	.059 (.057-.061)	.065
2. Exclusion of non-signif control variable (age)	2805.352	719*	.943	.056 (.054-.058)	.063
3. Exclusion of all non-signif variables (age + health perception + stress)	1760.786	312*	.954	.071 (.067-.074)	.073

*p value= .000

Table 2. Standardized coefficient, standard error, and significance level of each pathway in models 1 and 3 (see Table 3).

	Model 1			Model 3		
	Estimate	Std error	P value	Estimate	Std error	P value
**Regressions**						
PRS ← Safety (unsafe)	-.548	.104	.000	-.547	.104	.000
PRS ← Naturalness	.371	.079	.000	.369	.079	.000
PRS ← Management	.254	.056	.000	.255	.056	.000
PRS ← Soundscape	.183	.047	.001	.192	.047	.000
PRS ← Income (high)	.111	.076	.000	.109	.075	.000
PRS ← Sex (female)	.063	.075	.035	.060	.075	.044
PRS ← Stress perception	-.037	.084	.364	-	-	-
PRS ← Age	.011	.002	.696	-	-	-
PRS ← Health perception	-.008	.091	.857	-	-	-
**Correlations**						
Soundscape ↔ Naturalness	.627	.099	.000	.628	.098	.000
Management ↔ Naturalness	.760	.108	.000	.762	.108	.000
Health ↔ Stress	.531	.022	.000	-	-	-
Safety(unsafe)↔Sex(female)	.116	.007	.000	.116	.007	.000
Safety(unsafe)↔Income(high)	-.127	.007	.000	-.126	.007	.000

Table 3. List of variables originally included in each of the perceptions models before selection for final model analysis (according to multicollinearity).

Naturalness perception = landuse + proportion green 1km + area + perimeter-area ratio + proportion canopy[1] + proportion open vegetation[2] + tree species + tree species/ha + proportion native trees + bushes richness + proportion native bushes + water score + topography + number of habitats + understorey + bird species[3]

Management perception = proportion open vegetation + tree species + tree species/ha + proportion native trees + exotic trees[4] + bushes richness[5] + proportion native bushes + understorey + cleanliness + vandalism

Soundscape perception = landuse + proportion green 1km + area + perimeter-area ratio + proportion canopy + proportion open veg + water score + topography + number of habitats + bird species

Safety perception = landuse + proportion green 1km + area + perimeter-area ratio + proportion canopy + topography + understorey + vandalism

[1] Proportion canopy: consider categories 2, 3, 5, 9, 10, 13 of the Digital Mapping of Sao Paulo Vegetation Cover.

[2]Proportion open vegetation: consider categories 11 and 14 of the Digital Mapping of Sao Paulo Vegetation Cover.

[3]Bird species: data from Sao Paulo Municipality Wildlife Inventory.

[4]Exotic trees: Number of tree species classified as exotic.

[5]Bushes richness: Number of bushes species.

Table 4. Parameters used to check the validity of scales.

Scale	Mean (SD)	Cronbach's α	χ^2 (df)	CFI	RMSEA	SRMR
Perceived Restorativeness Scale	4.02 (1.13)	.92	133.453 (87)*	.997	.023 (.015-.031)	.041
Being away (5 items)	4.14 (1.22)	.80				
Fascination (5 items)	3.83 (1.30)	.83				
Compatibility (5 items)	4.10 (1.26)	.84				
Perceived Stress Scale (10 items)	16.99 (6.88)	.81	144.116 (35)*	.968	.056 (.047-.066)	.057
Perceived health		.76	648.849 (3)*	.999	.0001	.0001
General health (1 item)	2.75 (0.98)					
Mental health (1 item)	2.75 (1.04)					
Wellbeing (1 item)	2.71 (0.97)					
Setting perceptions			74.104 (24)*	.991	.046 (.034-.058)	.043
Soundscape (3 items)	3.31 (1.37)	.73				
Management (3 items)	3.31 (1.66)	.88				
Naturalness (3 items)	3.09 (1.34)	.70				

* $p \leq .001$
