# Supplementary material for: Urban park qualities driving visitors mental well-being and wildlife conservation in a Neotropical megacity
Source: Sci Rep. 2024 Feb 28;14:4856. doi: 10.1038/s41598-024-55357-2 (PMC10902329; doi:10.1038/s41598-024-55357-2)
Supplement: Supplementary file 2 — Supplementary Tables. [file 41598_2024_55357_MOESM2_ESM.docx]

Supplementary material

Table 1. Fit indices of the structural equation models.

| Model | χ^2^ | df | CFI | RMSEA | SRMR |
| --- | --- | --- | --- | --- | --- |
| 1. Full model (Perceptions + controls) | 3176.218 | 755* | .935 | .059 (.057-.061) | .065 |
| 1. Exclusion of non-signif control variable (age) | 2805.352 | 719* | .943 | .056 (.054-.058) | .063 |
| 1. Exclusion of all non-signif variables (age + health perception + stress) | 1760.786 | 312* | .954 | .071 (.067-.074) | .073 |

*p value= .000

Table 2. Standardized coefficient, standard error, and significance level of each pathway in models 1 and 3 (see Table 3).

|  | Model 1 | | |  | Model 3 | | |
| --- | --- | --- | --- | --- | --- | --- | --- |
|  | Estimate | Std error | P value |  | Estimate | Std error | P value |
| **Regressions** |  |  |  |  |  |  |  |
| PRS ← Safety (unsafe) | -.548 | .104 | .000 |  | -.547 | .104 | .000 |
| PRS ← Naturalness | .371 | .079 | .000 |  | .369 | .079 | .000 |
| PRS ← Management | .254 | .056 | .000 |  | .255 | .056 | .000 |
| PRS ← Soundscape | .183 | .047 | .001 |  | .192 | .047 | .000 |
| PRS ← Income (high) | .111 | .076 | .000 |  | .109 | .075 | .000 |
| PRS ← Sex (female) | .063 | .075 | .035 |  | .060 | .075 | .044 |
| PRS ← Stress perception | -.037 | .084 | .364 |  | - | - | - |
| PRS ← Age | .011 | .002 | .696 |  | - | - | - |
| PRS ← Health perception | -.008 | .091 | .857 |  | - | - | - |
| **Correlations** |  |  |  |  |  |  |  |
| Soundscape ↔ Naturalness | .627 | .099 | .000 |  | .628 | .098 | .000 |
| Management ↔ Naturalness | .760 | .108 | .000 |  | .762 | .108 | .000 |
| Health ↔ Stress | .531 | .022 | .000 |  | - | - | - |
| Safety(unsafe)↔Sex(female) | .116 | .007 | .000 |  | .116 | .007 | .000 |
| Safety(unsafe)↔Income(high) | -.127 | .007 | .000 |  | -.126 | .007 | .000 |

Table 3. List of variables originally included in each of the perceptions models before selection for final model analysis (according to multicollinearity).

**Naturalness perception** = landuse + proportion green 1km + area + perimeter-area ratio + proportion canopy^1^ + proportion open vegetation^2^ + tree species + tree species/ha + proportion native trees + bushes richness + proportion native bushes + water score + topography + number of habitats + understorey + bird species^3^

**Management perception** = proportion open vegetation + tree species + tree species/ha + proportion native trees + exotic trees^4^ + bushes richness^5^ + proportion native bushes + understorey + cleanliness + vandalism

**Soundscape perception** = landuse + proportion green 1km + area + perimeter-area ratio + proportion canopy + proportion open veg + water score + topography + number of habitats + bird species

**Safety perception** = landuse + proportion green 1km + area + perimeter-area ratio + proportion canopy + topography + understorey + vandalism

^1^ Proportion canopy: consider categories 2, 3, 5, 9, 10, 13 of the Digital Mapping of Sao Paulo Vegetation Cover.

^2^Proportion open vegetation: consider categories 11 and 14 of the Digital Mapping of Sao Paulo Vegetation Cover.

^3^Bird species: data from Sao Paulo Municipality Wildlife Inventory.

^4^Exotic trees: Number of tree species classified as exotic.

^5^Bushes richness: Number of bushes species.

Table 4. Parameters used to check the validity of scales.

| Scale | Mean (SD) | Cronbach’s α | χ^2^ (df) | CFI | RMSEA | SRMR |
| --- | --- | --- | --- | --- | --- | --- |
| *Perceived Restorativeness Scale* | 4.02 (1.13) | .92 | 133.453 (87)* | .997 | .023 (.015-.031) | .041 |
| Being away (5 items) | 4.14 (1.22) | .80 |  |  |  |  |
| Fascination (5 items) | 3.83 (1.30) | .83 |  |  |  |  |
| Compatibility (5 items) | 4.10 (1.26) | .84 |  |  |  |  |
|  |  |  |  |  |  |  |
| *Perceived Stress*  *Scale (10 items)* | 16.99 (6.88) | .81 | 144.116 (35)* | .968 | .056 (.047-.066) | .057 |
|  |  |  |  |  |  |  |
| *Perceived health* |  | .76 | 648.849 (3)* | .999 | .0001 | .0001 |
| General health (1 item) | 2.75 (0.98) |  |  |  |  |  |
| Mental health (1 item) | 2.75 (1.04) |  |  |  |  |  |
| Wellbeing (1 item) | 2.71 (0.97) |  |  |  |  |  |
|  |  |  |  |  |  |  |
| *Setting perceptions* |  |  | 74.104 (24)* | .991 | .046 (.034-.058) | .043 |
| Soundscape (3 items) | 3.31 (1.37) | .73 |  |  |  |  |
| Management (3 items) | 3.31 (1.66) | .88 |  |  |  |  |
| Naturalness (3 items) | 3.09 (1.34) | .70 |  |  |  |  |

* p≤ .001
